# Broca Pars Triangularis Constitutes a “Hub” of the Language-Control Network during Simultaneous Language Translation

**DOI:** 10.3389/fnhum.2016.00491

**Published:** 2016-09-29

**Authors:** Stefan Elmer

**Affiliations:** Auditory Research Group Zurich, Division Neuropsychology, Institute of Psychology, University of ZurichZurich, Switzerland

**Keywords:** executive functions, simultaneous interpreters, fMRI, single-subject analyses, overt speech

## Abstract

Until now, several branches of research have fundamentally contributed to a better understanding of the ramifications of bilingualism, multilingualism, and language expertise on psycholinguistic-, cognitive-, and neural implications. In this context, it is noteworthy to mention that from a cognitive perspective, there is a strong convergence of data pointing to an influence of multilingual speech competence on a variety of cognitive functions, including attention, short-term- and working memory, set shifting, switching, and inhibition. In addition, complementary neuroimaging findings have highlighted a specific set of cortical and subcortical brain regions which fundamentally contribute to administrate cognitive control in the multilingual brain, namely Broca's area, the middle-anterior cingulate cortex, the inferior parietal lobe, and the basal ganglia. However, a disadvantage of focusing on group analyses is that this procedure only enables an approximation of the neural networks shared within a population while at the same time smoothing inter-individual differences. In order to address both commonalities (i.e., within group analyses) and inter-individual variability (i.e., single-subject analyses) in language control mechanisms, here I measured five professional simultaneous interpreters while the participants overtly translated or repeated sentences with a simple subject-verb-object structure. Results demonstrated that pars triangularis was commonly activated across participants during backward translation (i.e., from L2 to L1), whereas the other brain regions of the “control network” showed a strong inter-individual variability during both backward and forward (i.e., from L1 to L2) translation. Thus, I propose that pars triangularis plays a crucial role within the language-control network and behaves as a fundamental processing entity supporting simultaneous language translation.

## Introduction

Simultaneous language translation constitutes a complex linguistic task that strongly relies on executive functions. In fact, this specific task implies attentive listening to the input language while at the same time maintaining the source information in short-term memory, articulating in a target language (working memory and language switching), controlling the output language (divided attention), and inhibiting the articulatory codes of the input language (Elmer, 2012). Previous structural (Elmer et al., 2011a, 2014a) and functional neuroimaging studies performed with professional simultaneous interpreters (SIs) (Rinne et al., 2000; Elmer et al., 2011b; Hervais-Adelman et al., 2015a) and multilingual subjects (Price et al., 1999; Rodriguez-Fornells et al., 2006; Abutalebi and Green, 2007; Hervais-Adelman et al., 2015b), have identified Broca's area, the caudate nuclei, the middle-anterior cingulate cortex, as well as the inferior parietal lobe as being part of the language-control network. Notably, the same brain regions have also been shown to be functionally (Elmer et al., 2011b; Hervais-Adelman et al., 2015a) and structurally (Elmer et al., 2011a, 2014a) altered in professional SIs compared to multilingual control subjects. In particular, professional SIs demonstrated reduced gray matter volume in the left middle-anterior cingulate gyrus, bilateral pars triangularis, left pars opercularis, bilateral middle part of the insula, and in the left supramarginal gyrus (Elmer et al., 2014a). Interestingly, gray matter volume in left pars triangularis, right pars opercularis, middle-anterior cingulate cortex, and in the bilateral caudate nuclei has also been shown to correlate fairly well with the cumulative number of interpreting hours (Elmer et al., 2014a). In addition, SIs have previously been shown to be characterized by reduced fractional anisotropy in fiber tracts encompassing the left anterior insula, the right inferior parietal lobe, the dorsal part of the right caudate nucleus as well as the cingulum. Finally, previous fMRI studies reported a training-related reduced recruitment of the right caudate nucleus during simultaneous language translation (Hervais-Adelman et al., 2015a) as well as a displacement of attentional functions from frontal to parietal brain sites (Elmer et al., 2011b) in SIs compared to multilingual control subjects.

Certainly, it is important to mention that comparable effects have previously already been extensively described in bilinguals and multilinguals (Price et al., 1999; Abutalebi et al., 2000; Rodriguez-Fornells et al., 2002; Mechelli et al., 2004; Abutalebi and Green, 2007; Hernandez, 2009; Zou et al., 2012a,b; Della Rosa et al., 2013), leading to suggest a general organizational principle underlying language control mechanisms. Importantly, the prominent engagement of cognitive control mechanisms necessarily required to manage multiple languages with one brain has also been shown to be associated with behavioral advantages in a variety of cognitive domains, including attention (Bialystok et al., 2004; Costa et al., 2008), working memory (Morales et al., 2013), set shifting (Festman and Münte, 2013), and inhibition (Bialystok et al., 2004; Festman et al., 2010).

A main drawback of previous studies is that most of them exclusively focused on group analyses. Even though there is no doubt that group analyses constitute a powerful approach for inferring generalization of brain functions within a specific population, by using this procedure it results difficult to ensure that a particular brain region is consistently activated above threshold in each single participant. In other words, this procedure is especially powerful for estimating functional commonalities while at the same time neglecting inter-individual differences. This can be especially problematic when it comes to evaluate brain activity in small samples of subjects or even when it comes to evaluate the contribution of higher cognitive functions. In fact, function and anatomy of brain areas at the top of the hierarchical organization are more variable across individuals compared to those involved in more basic perceptual and motor processes (Gordon et al., 2015). Otherwise, single-subject analyses have the drawback of rendering difficult the comparability of results across subjects, for example when it comes to associate activity originating from slightly different clusters within a particular brain region with the underling functions. Consequently, a combination of single-subject- and group analyses constitutes an interesting approach that enables a more holistic description of brain functions, especially in small samples of subjects.

In the current fMRI study, I combined single-subjects and group analyses, and addressed inter-individual commonalities as well as differences in the recruitment of the language-control network in five professional simultaneous interpreters while the subjects performed a simultaneous language translation task or overtly repeated simple auditory presented sentences. Based on previous work (Abutalebi and Green, 2007; Elmer et al., 2014a), statistical analyses were restricted to a specific set of a-priori defined brain regions that have repeatedly been shown to support cognitive control mechanisms (Abutalebi and Green, 2007) and to be altered as a function of interpreting training (Abutalebi and Green, 2007; Elmer et al., 2011a, 2014a), namely bilateral Broca's area, the inferior parietal lobe, the middle-anterior cingulate cortex, the caudate nuclei, and the insula.

Since its discovery in the middle of the nineteenth century, Broca's region has attracted considerable attention, especially in the domain of speech and language processing (Price, 2000, 2012; Bookheimer, 2002; Fiebach et al., 2005; Friederici, 2006). Broca's region is situated in the ventral-posterior part of the inferior frontal gyrus and can be subdivided into a rostral and caudal part, namely pars triangularis and pars opercularis, respectively. From a histological perspective, pars opercularis is a dysgranular area characterized by a sparse pronounced fourth layer. Therefore, this brain region is often considered to be part of motor territory (Amunts et al., 1999; Anwander et al., 2007; Kelly et al., 2010) and to constitute a higher instance supporting speech processing, articulation as well as phonological processing (Eickhoff et al., 2009; Price, 2012). By contrast, pars triangularis is a granular area which has the same histological architecture as other brain regions situated in the prefrontal cortex (Amunts et al., 1999; Anwander et al., 2007; Kelly et al., 2010) and sub-serving higher cognitive functions (Amunts et al., 1999). Even though pars triangularis has repeatedly been associated with linguistic processes, including syntax and semantic (Thompson et al., 1999; Fiebach et al., 2005; Friederici, 2006; Tyler et al., 2011; Bornkessel-Schlesewsky and Schlesewsky, 2013; Friederici and Gierhan, 2013), its precise functional role still remains somewhat controversial.

In the last decades, the functional role of Broca's area (pars opercularis and triangularis) has fundamentally been revised. In fact, nowadays there is a growing body of evidence indicating that Broca's area is not exclusively a speech- and language-selective region but sub-serves a variety of cognitive functions (Fedorenko et al., 2012; Fedorenko, 2014). For example, Eickhoff and colleagues performed a meta-analysis of neuroimaging studies acquired during a variety of verbal fluency tasks and revealed that pars opercularis more likely constitutes the final stage of word retrieval from memory rather than supporting articulation *per se* (Eickhoff et al., 2009). Accordingly, activity in pars triangularis has previously repeatedly been observed while performing different cognitive tasks, like language switching, attention, and inhibition (Price et al., 1999; Fuster, 2001; Rodriguez-Fornells et al., 2002, 2006; Rogalsky and Hickok, 2011; Fedorenko et al., 2012; Santi and Grodzinsky, 2012). Furthermore, there is even a convergent body of data indicating that the same area supports verbal working memory functions during both single-words (Chein et al., 2002) or sentence processing (Rogalsky and Hickok, 2011), especially in high-load conditions (Caplan and Waters, 1999). Notably, since language-selective and domain-general regions are assumed to lay side by side within Broca's area (Fedorenko et al., 2012), this specific brain region constitutes the most eligible candidate for being considered as a “hub” region within the language-control network.

Since the pivotal work of Dronkers (1996) and Wise et al. (1999), the left anterior insula has repeatedly been identified as being crucially involved, among other functions (Flynn et al., 1999; Sterzer and Kleinschmidt, 2010), in articulation and phonation (Ackermann and Riecker, 2010; Kurth et al., 2010; Baldo et al., 2011), regulation of ventilation during speech production (Dronkers, 1996; Ackermann and Riecker, 2010; Baldo et al., 2011), and sensory-to-motor coupling mechanisms (Mutschler et al., 2007, 2009), the latter contributing to the transformation of phonetic representations into articulatory codes. Previous fMRI studies have reported both white- (Elmer et al., 2011a) and gray (Elmer et al., 2014a) matter changes in the left anterior insula in SIs compared to multilinguals, and the same region has been shown to be structurally altered in French subjects who learned to pronounce Persian consonants (Golestani and Pallier, 2007; Golestani et al., 2007). From an anatomical perspective, the medial border of the insula adjoins to the extreme capsule, a fiber bundle that runs to Broca's area, the planum temporale, as well as to the brain stem (Ozaki et al., 1986), and via the latter rely station, to the primary motor cortex and the cerebellum. Consequently, the insula may sub-serve the integration of information between the auditory-related cortex and the motor system by supporting sensory-motor coupling mechanisms (Mutschler et al., 2007, 2009), for example, during the adjustment of articulation through auditory feedback (Berken et al., 2015). Finally, a previous dynamic causal modeling-based meta-analysis of Eickhoff et al. (2009) provided clear evidence for a functional architecture featuring the insula in a serial position between pars opercularis and the cerebellum/basal ganglia, from where the information flow converges onto the premotor- and motor cortex. In this context, it is assumed that the left anterior insula receives information about phonetic representations from Broca's regions and the auditory-related cortex, and translates it into vocal tract motor programs (Eickhoff et al., 2009).

The caudate nuclei are richly interconnected with the prefrontal cortex via cortico-striatal loops (Leh et al., 2007). Even though these nuclei have repeatedly been associated with the control of voluntary movements (Obeso et al., 2000) and speech articulation (Eickhoff et al., 2009), recent clinical (Wang et al., 2013), and neuroimaging (Li et al., 2015) data clearly emphasize a further functional contribution of the head of caudate to language control mechanisms. In particular, the caudate nuclei have been proposed to be involved in monitoring and controlling the language in use (Crinion et al., 2006). This perspective emerges in a salient manner from a previous meta-analysis of neuroimaging data (Luk et al., 2012), where Luk and colleagues evaluated functional responses in bilinguals during voluntary language switching.

The middle-anterior part of the cingulate cortex is a heterogeneous brain region (Bush et al., 2000) underlying a variety of cognitive functions, like attention, conflict monitoring, error detection, inhibition as well as language switching mechanisms (Bush et al., 2000; Abutalebi and Green, 2007; Wang et al., 2007; Moritz-Gasser and Duffau, 2009). Currently, available data indicate functional (Abutalebi et al., 2008; Luk et al., 2012) and structural (Abutalebi et al., 2012; Zou et al., 2012b) differences in this brain region in bilinguals compared to monolinguals or even between professional SIs and multilingual control subjects (Elmer et al., 2010, 2014a).

The inferior parietal lobe (i.e., angular and supramarginal gyrus) has previously been proposed to be part of the language control network (Abutalebi and Green, 2007) and shown to be functionally and structurally altered as a function of both bilingualism (Mechelli et al., 2004; Della Rosa et al., 2013) and language expertise (Elmer et al., 2011b, 2014a). Furthermore, in bilinguals, gray matter volume in the inferior parietal lobe was found to correlate with L2 proficiency (Mechelli et al., 2004; Della Rosa et al., 2013) as well as with age of L2 acquisition (Mechelli et al., 2004). This posterior brain region sub-serves a heterogeneity of cognitive functions including attention (Elmer et al., 2011b; Karnath and Rorden, 2012; Alho et al., 2014), working memory, and short-term memory (Buchsbaum et al., 2002; Schulze and Koelsch, 2012; Herman et al., 2013), all functions that have been documented to be influenced by language experience and expertise (Bialystok et al., 2004; Abutalebi and Green, 2007; Bialystok, 2009). Complementary findings also reliably converge to the notion that the inferior parietal lobe, especially in the left hemisphere, supports linguistic functions, and phonetic processing (Ruff et al., 2003; Turkeltaub and Coslett, 2010).

## Materials and methods

### Participants

Five professional SIs that exclusively translate from German (L2) to Italian (L1) participated in the study. In order to better address whether commonalities as well as intra-individual differences are influenced by experience and age, I explicitly selected two subjects with comparable age and number of training years (i.e., subject 4 and 5, age = 46 and 50, training years = 22), two subjects of the same age cohort but differing in training (i.e., subject 1 and 2, age = 31 and 33 years, training years = 4 and 8, respectively), as well as a control subject (i.e., subject 3, age = 38, training years = 14) differing from the other subjects regarding both age and years of training. All subjects confirmed to have a very good to excellent proficiency in the two languages tested (i.e., L1 = Italian and L2 = German) as well as an intact audiological status. None of the control subjects grew up in a bilingual context, all participants were consistent right-handers (Annett scores: S1 11/12; S2 12/12; S3 12/12; S4 12/12; S5 12/12; the score was calculated based on right or not right responses, none of the participants reported ambidexterity) according to Annett's questionnaire (Annett, 1970), had a comparable level of education (i.e., university degree), reported no past or current neurological, psychiatric, or neuropsychological problems, and denied illegal medication. Subjects were paid for participation, the local ethics committee (Zurich, Switzerland) approved the study, and written informed consent was obtained from all participants. Table 1 provides an overview of the autobiographical data of the five SIs investigated in the present study.

**Table 1:** **Overview of the autobiographical data of the participants**.

**Subject**	**Age**	**A language**	**B language**	**C language**	**Years of training**
1	31	Italian		German, French, English	4
2	33	Italian	German	French, English	8
3	38	Italian		German, French, Spanish, English	14
4	46	Italian	French, German	English	22
5	50	Italian		German, English, French	22

*A language = mother tongue, B language = foreign language (high proficiency), C language = foreign language (good proficiency)*.

### Task

In the MRI environment, subjects heard short German and Italian sentences (totally 80 for each language). All sentences consisted of a subject-verb-object structure, had a mean duration of 1.75 s (Italian = 1.70; German = 1.85), were matched for word frequency according to the Leipzig corpora (http://corpora.uni-leipzig.de/), and double checked by a professional linguist (D.W.). In the present work, I deliberately choose simple discourse sentences in order to minimize interactions between cognitive load and experience while at the same time focusing on brain regions essentially involved in simultaneous language translation. During scanning, the Italian and German sentences were presented in a pseudo-randomized order, and according to a specific visual cue that was simultaneously presented with the onset of the sentences (i.e., star or cloud), the subjects were instructed to repeat them aloud (i.e., shadowing, i.e., 40 German and 40 Italian sentences) or simultaneously translate them (i.e., 40 German and 40 Italian sentences). During scanning, the participants were instructed to keep their eyes open and to focus on the fixation cross presented on the screen. The auditory stimuli were jittered with an ISI corresponding to 2–5 repetition times (TRs) and presented in the context of four consecutive runs, each of them lasting about 12 min. During scanning, the participants were instructed to press a response button when starting and finishing shadowing and translation. Table 2 provides an example of the stimulus material used.

**Table 2:** **Example of German and Italian sentences with a subject-verb-object structure**.

**Subject**	**Verb**	**Objekt**
Der Mann	grüsst	eine Nachbarin
Il maestro	assegna	un compito

### fMRI data acquisition and preprocessing

Binaural auditory stimuli were presented by a digital playback system and included a high-frequency shielded transducer system. The acoustic transmission system included a piezoelectric loudspeaker enabling the transmission of strong sound pressure levels (105 dB) with excellent attenuation characteristics (Jäncke et al., 2002). The approximate delivered intensity level in the scanner was about 90 dB. These loudspeakers were embedded in tightly occlusive headphones, allowing unimpeded conduction of the stimulus with good suppression of ambient scanner noise by about 20 dB. The headphones we used for the experiment had a frequency response ranging from 100 to 16 KHz. Additionally, noise-protection ear plugs within the loudspeakers provided an additional noise attenuation of about 15–20 dB, resulting in a total noise attenuation of 35–40 dB. The acoustic transmission system allowed stimulation of acoustic stimuli with relatively few distortions.

A Philips Intera 3-T whole-body MR unit (Philips Medical Systems, Best, Netherlands) equipped with an 8-channel Philips SENSE head coil was used to acquire fMRIs at the University Hospital, Zurich. Functional data were obtained from 320 whole-head scans per run using a Sensitivity Encoded (SENSE) single-shot echo-planar imaging (EPI) technique (TA/TR = 2000 ms, time echo = 35 ms, flip angle = 78°, field of view = 220 mm, acquisition matrix = 80 × 80, 30 transverse slices, voxel size = 1.72 × 1.72 × 4.00 mm).

MRI data analysis was performed by using MATLAB 2013b (Mathworks Inc., Natick, Massachusetts) and the SPM12 software package (Institute of Neurology, London, UK). All images were realigned to the first image of each run, spatially normalized into standard stereotactic MNI space (EPI template provided by the Montreal Neurological Institute), interpolated to a voxel size of 2.00 × 2.00 × 2.00 mm, and spatially smoothed using a 8-mm full-with a half-maximum Gaussian kernel.

### fMRI analyses

Statistical analysis was based on the general linear model (GLM). Due to the experimental design, event-related analyses were conducted. The standardized canonical hemodynamic response was applied to model the blood oxygen level–dependent response to each of the heard (i.e., Italian and German) and spoken (i.e., shadowing and translation) sentences. In addition, the behavioral responses (i.e., button press at the beginning and at the end of articulation) were modeled as events, and movement (i.e., pitch, roll, and jaw) correction parameters were modeled as multiple regressors.

On the first-level analysis, the comparisons of interest, namely L2 to L1 translation (i.e., backward translation) vs. L2 shadowing and L1 to L2 (i.e., forward translation) translation vs. L1 shadowing, were implemented as linear contrasts. The resulting set of voxel values for the contrast of interest constitutes a statistical parametric map of the single-subjects T-statistic. In the present work, I consciously abstained for contrasting L2 to L1 translation vs. L1 shadowing as well as L1 to L2 translation vs. L2 shadowing. In fact, different language inputs (i.e., L1 and L2) have an influence on the cognitive demands necessary for processing the native and non-native languages. Consequently, by maintaining the input language constant this specific confound is not present. Group analyses were performed by means of one-sample *t*-tests based on first-level single-subjects contrasts. Since the analyses were restricted (both first- and second-level) on a specific set of a-priori defined regions of interest (i.e., ROIs), all results are reported at a threshold of *p* < 0.01 and a voxel extent threshold of 10 voxels (uncorrected). The ROIs were choosen according to previous literature on cognitive control mechanisms in bilinguals (Abutalebi and Green, 2007) and SIs (Elmer et al., 2011a, 2014a), and consisted of the following brain regions: Broca's area (pars triangularis and opercularis), middle-anterior cingulate gyrus, caudate nuclei, supramarginal and angular gyrus, and middle-anterior insula. All ROIs were added into a single, composite brain mask image and explicitly applied to restrict the statistical analysis to the voxels within this mask. The first five ROIs were taken from the functional-anatomical framework proposed by Abutalebi and Green (2007), and originated from the Harvard–Oxford cortical and subcortical structural atlases as implemented in the FSL software package (http://fsl.fmrib.ox.ac.uk/fsl/fslwiki/Atlases). The insula was selected based on previous neuroimaging studies performed with SIs (Elmer et al., 2011a, 2014a) and also originated from the Harvard–Oxford cortical structural atlas. All ROIs were threshold at 30% probability.

## Results

### Behavioral data

The evaluations of the behavioral data indicated that all subjects were able to shadow and translate all sentences without problems. In addition, all participants confirmed that the task was very simple in comparison to their daily work situation as SIs. Overall, all subjects were slightly faster [*t*_(4)_ = 0.946, *p* = 0.398, n.s.] in shadowing Italian (mean = 2.64 s, *SD* = 0.25) compared to German (mean = 2.70, *SD* = 0.29), as well as faster in translating [*t*_(4)_ = 4.843, *p* = 0.008) from L1 to L2 (mean = 2.78, *SD* = 0.33) compared to the backward translation (i.e., L2 to L1, mean = 3.13, *SD* = 0.26). The longer shadowing time in German as well as the longer L2 to L1 translation time is possibly to attribute to the fact that the German sentences were overall longer than the Italian ones. Otherwise, it is even possible that the Italian mother tongue of the participants may lay at the basis of this effect.

### Single-subject analyses

**Figure 1:**
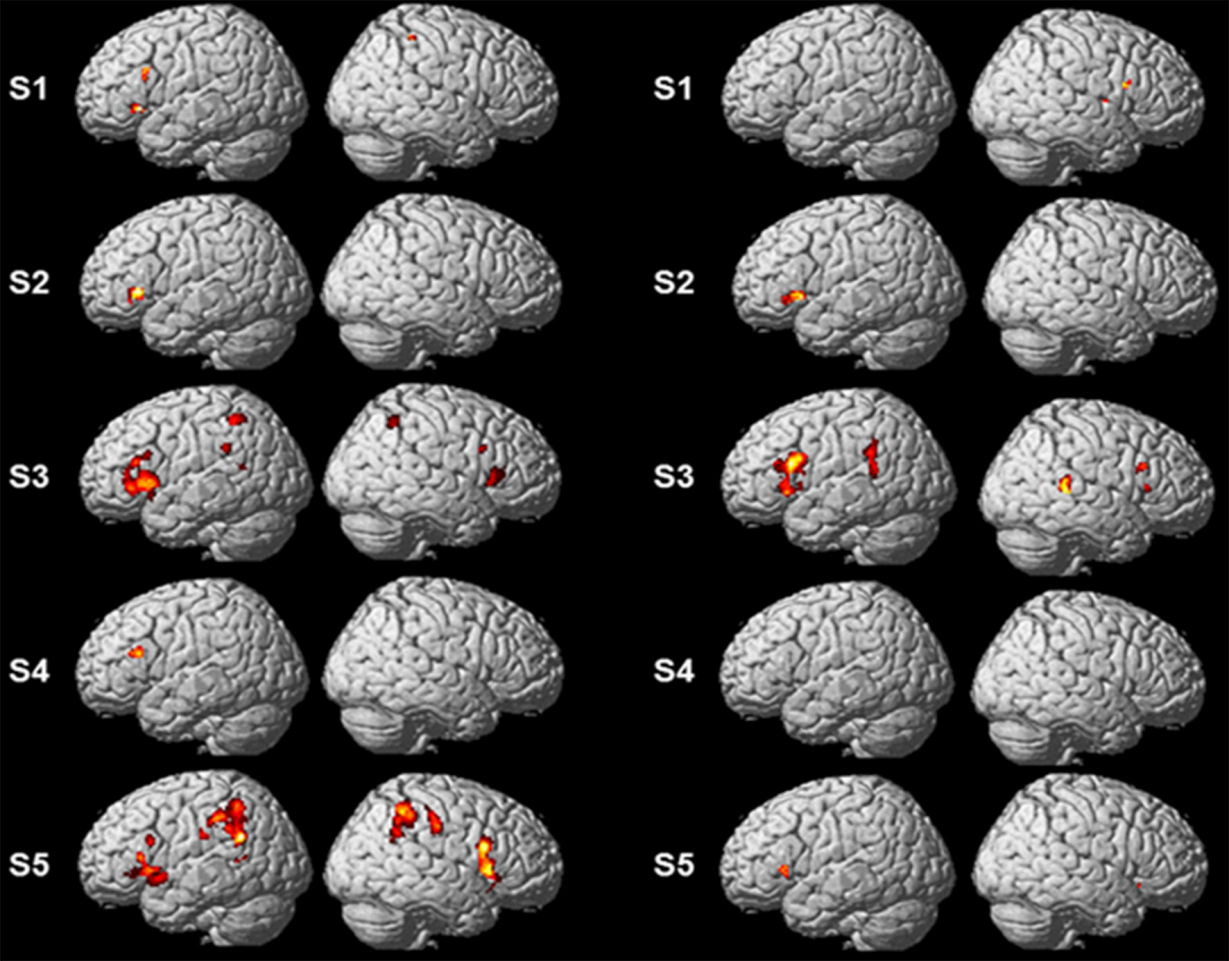
**Results of the single-subject analyses**. Significant results for each subject (S1–S5) are shown on rendering surfaces for both the linear contrasts “translation L2 to L1” vs. “shadowing L2” (left side) and “translation L1 to L2” vs. “shadowing L1” (right side).

**Figure 2:**
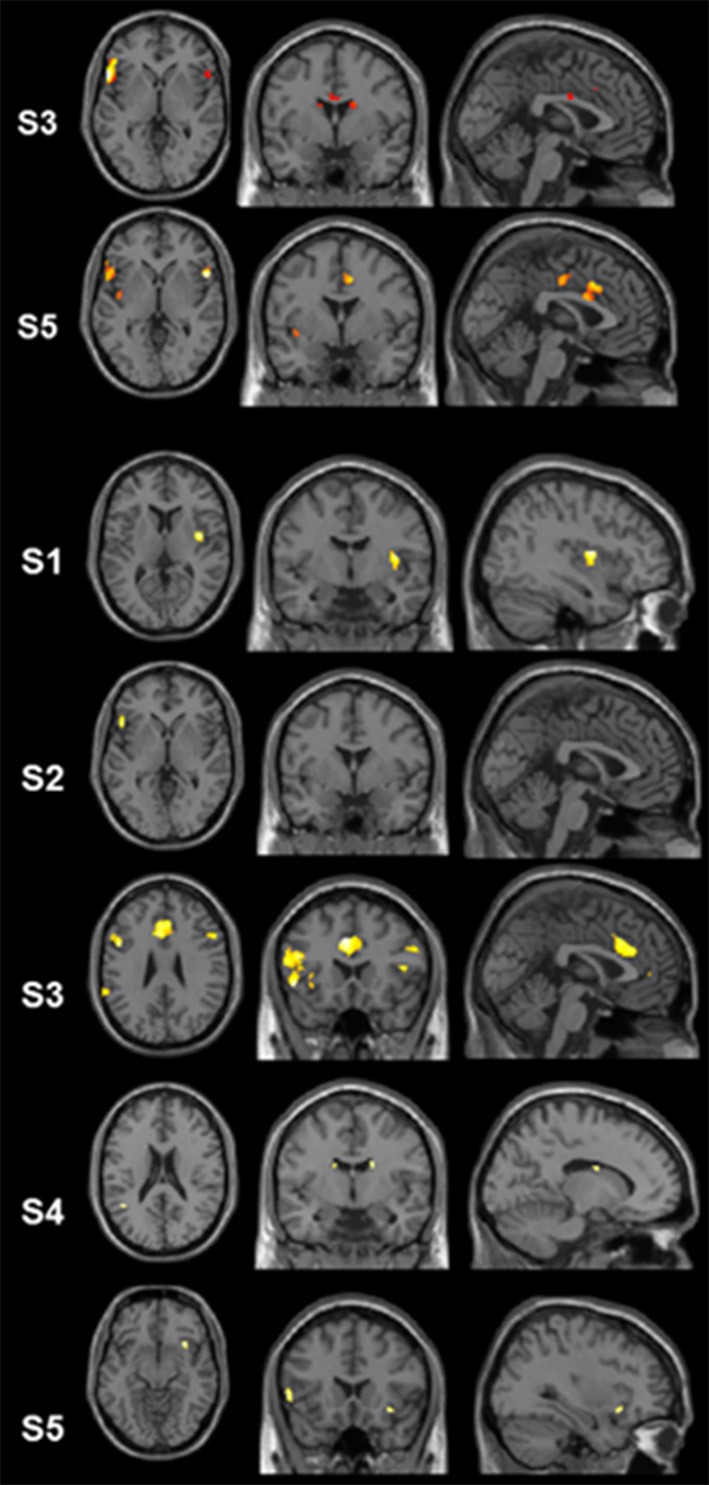
**Results of the single-subject analyses**. Significant results for the linear contrasts “translation L2 to L1” vs. “shadowing L2” (first three rows, S3, S5, S1) and “translation L1 to L2” vs. “shadowing L1” (last four rows, S2–S5) are depicted on transversal (left), coronal (middle), and sagittal (rigth) brain slices. The intersecting planes correpond to the coordinates depicted in Tables 3, 4.

Single-subject analyses consistently revealed increased activity in left pars triangularis in all five subjects during backward translation. In addition, during the same condition results revealed a huge inter-individual variability in all other regions of the language-control network (left column of Figures 1, 2 and Table 3). By contrast, during forward translation single-subject analyses did not reveal such a huge overlap across subjects but rather high variability (right column of Figures 1, 2 and Table 4).

**Table 3:** **Peak maxima of each subject (S1–S5) for the contrast “translation L2 to L1” vs. “shadowing L2.**”

**Subject**	***P*-value (uncorrected)**	***T*-value**	**MNI coordinates**			**Hemisphere**	**Brain region**	**Cluster size**
			***x***	***y***	***z***			
S1	0.001	3.25	−50	24	−4	L	Inferior frontal gyrus, p. Triangularis	32
	0.001	3.12	−38	22	−2	L	Inferior frontal gyrus, p. Triangularis	19
	0.001	3.02	−54	18	30	L	Inferior frontal gyrus, p. Triangularis	35
	0.004	2.63	44	−42	56	R	Supramarginal gyrus	11
S2	0.00001	3.57	−48	26	0	L	Inferior frontal gyrus, p. Triangularis	85
S3	0.00001	6.7	−56	16	0	L	Inferior frontal gyrus, p. Opercularis & Triangularis	543
	0.00001	3.87	−50	−50	56	L	Supramarginal gyrus	111
	0.00001	3.6	52	−54	54	R	Supramarginal gyrus	47
	0.00001	3.51	−42	18	−4	L	Inferior frontal gyrus, p. Triangularis	24
	0.00001	3.36	52	14	32	R	Inferior frontal gyrus, p. Opercularis	26
	0.00001	3.3	−4	−4	30	L	Middle cingulate cortex	40
	0.001	3.23	−2	22	36	L	Middle-anterior cingulate cortex	13
	0.001	3.2	16	−2	24	R	Caudate nucleus	37
	0.001	3.13	−62	−40	32	L	Supramarginal gyrus	38
	0.002	2.89	−16	−18	24	L	Caudate nucleus	10
	0.002	2.85	54	20	2	R	Inferior frontal gyrus, p. Triangularis	103
	0.003	2.8	−12	0	22	L	Caudate nucleus	10
	0.004	2.64	−50	−54	16	L	Angular gyrus	12
S4	0.0001	4	−54	24	22	L	Inferior frontal gyrus, p. Triangularis	66
S5	0.0001	5.03	54	16	0	R	Inferior frontal gyrus, p. Opercularis	401
	0.0001	4.36	−52	18	−4	L	Inferior frontal gyrus, p. Triangularis	178
	0.0001	4.15	−44	12	−8	L	Anterior insula	224
	0.0001	4.1	40	−42	48	R	Supramarginal gyrus	292
	0.0001	3.95	66	−22	38	R	Supramarginal gyrus	127
	0.0001	3.94	−40	−52	26	L	Angular gyrus	682
	0.0001	3.67	−4	14	38	L	Middle-anterior cingulate cortex	370
	0.0001	3.34	−62	−24	30	L	Supramarginal gyrus	42
	0.001	3.23	−56	18	24	L	Inferior frontal gyrus, p. Triangularis	29
	0.001	3.08	0	−12	44	R	Middle cingulate cortex	80
	0.001	3.04	−50	−50	8	L	Angular gyrus	13
	0.002	2.85	18	−16	24	R	Caudate nucleus	14
	0.002	2.84	58	−52	38	R	Supramarginal gyrus	91
	0.003	2.78	36	20	−10	R	Anterior insula	14

*P < 0.01 (uncorrected)*.

**Table 4:** **Peak maxima of each subject (S1–S5) for the contrast “translation L1 to L2” vs. “shadowing L1.**”

**Subject**	***P*-value (uncorrected)**	***T*-value**	**MNI coordinates**			**Hemisphere**	**Brain region**	**Cluster size**
			***x***	***y***	***z***			
S1	0.0001	3.73	40	−2	12	R	Middle insula	90
	0.005	2.58	56	12	20	R	Inferior frontal gyrus, p. Opercularis	11
S2	0.0001	4.07	−50	22	−4	L	Inferior frontal gyrus, p. Triangularis	70
S3	0.0001	3.78	−4	22	32	L	Middle-anterior cingulate cortex	477
	0.0001	3.52	−50	20	−4	L	Inferior frontal gyrus, p. Triangularis	493
	0.0001	3.35	56	−40	14	R	Angular gyrus	151
	0.001	3.11	−34	−12	10	L	Middle insula	15
	0.001	3.05	44	22	10	R	Inferior frontal gyrus, p. Triangularis	20
	0.001	2.97	−34	24	−4	L	Anterior insula	30
	0.002	2.97	38	−18	14	R	Posterior insula	25
	0.002	2.96	−62	−42	30	L	Supramarginal gyrus	131
	0.002	2.9	56	18	26	R	Inferior frontal gyrus, p. Opercularis	42
	0.002	2.87	10	40	10	R	Anterior cingulate cortex	42
S4	0.001	3.18	18	−10	26	R	Middle cingulate cortex	20
	0.001	3.04	−42	−50	20	L	Angular gyrus	15
	0.003	2.79	−14	−2	24	L	Caudate nucleus	12
S5	0.01	3.07	−56	24	6	L	Inferior frontal gyrus, p. Triangularis	38
	0.001	2.89	34	18	−12	R	Anterior insula	25

*P < 0.01 (uncorrected)*.

### Group analyses

**Figure 3:**
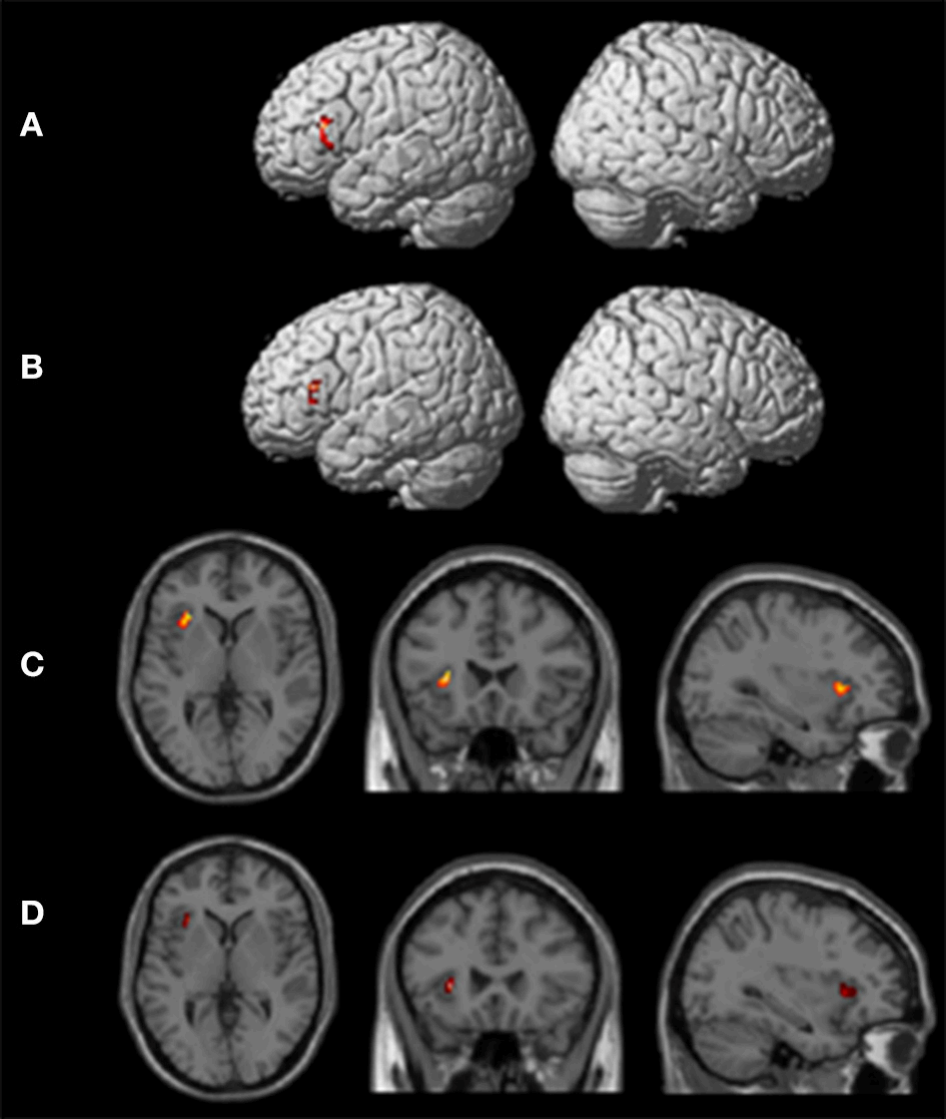
**Results of the group analyses**. Significant results for the the linear contrasts “translation L2 to L1” vs. “shadowing L2” **(A,B)** and “translation L1 to L2” vs. “shadowing L1” **(C,D)** are shown on rendering surfaces **(A,B)** as well as on transversal (left), coronal (middle), and sagittal (rigth) brain slices **(C,D)**. The intersecting planes correpond to the coordinates depicted in Table 5. **(A,C)** represent group analyses without covariates, **(B,D)** reflect the same contrast with age as covariate.

One-sample *t*-tests of first-level contrasts revealed a significant cluster situated within left pars triangularis during backward translation (Figure 3A and Table 5). By contrast, forward translation was associated with increased activity in a region situated within the left anterior insula (Figure 3C and Table 5). These results did not change when modeling age as a covariate (Figures 3B,D and Table 5).

**Table 5:** **Peak maxima of group results for the contrast “translation L2 to L1” vs. “shadowing L2” and “translation L1 to L2” vs. “shadowing L1” both with and without age as covariate**.

**Contrast**	**Covariate**	***P*-value (uncorrected)**	***T*-value**	**MNI coordinates**			**Hemisphere**	**Brain region**	**Cluster size**
				***x***	***y***	***z***			
L2 to L1 vs. shadowing L2	None	0.001	7.71	−46	24	20	Left	Inferior frontal gyrus, p. Triangularis	68
	Age	0.001	12.13	−56	26	16	Left	Inferior frontal gyrus, p. Triangularis	35
L1 to L2 vs. shadowing L1	None	0.0001	10.11	−30	22	6	Left	Anterior insula	92
	Age	0.0001	49.63	−30	24	4	Left	Anterior insula	62

*P < 0.01 (uncorrected)*.

## Discussion

### General discussion

Until now, only a few studies have addressed the functional and anatomical correlates of simultaneous language translation in professional- (Rinne et al., 2000; Proverbio et al., 2004, 2009; Elmer et al., 2010, 2011a,b, 2014a,b; Elmer, 2012) and trainee SIs (Hervais-Adelman et al., 2015a) or bilinguals (Price et al., 1999; Klein et al., 2006; Hervais-Adelman et al., 2015b). Furthermore, all previous studies focused on statistical analyses at the group-level while completely neglecting inter-individual variability in the recruitment of language-control networks. A second drawback is that most of these studies used complex and variable sentence structures that pose additional demands on executive functions potentially interacting with the core processes underlying simultaneous language translation. In addition, some studies investigated bilinguals (Klein et al., 2006; Price et al., 1999; Hervais-Adelman et al., 2015b) or trainee interpreting students (Hervais-Adelman et al., 2015a) instead of professional SIs, leading to unspecific results and putative confounds. Finally, it is noteworthy to mention that some authors even measured SIs who differed in the trained language direction, leading to contamination effects and resulting in limited explanatory power (Hervais-Adelman et al., 2015a).

In the present work, I used fMRI and measured five professional SIs while the subjects performed backward- and forward simultaneous translations of simple sentences with a subject-verb-object structure or simply repeated the heard sentences (i.e., shadowing). In addition, in order to capture both the brain regions essentially involved in administrating cognitive control mechanisms (i.e., “hub” areas) as well inter-individual differences (i.e., “supportive” areas), I combined single-subjects- and group analyses. Importantly, based on previous work indicating functional differences in the processing of different languages (i.e., perception and articulation) (Kim et al., 1997; Perani et al., 1998, 2003; Paulesu et al., 2000), here I exclusively measured SIs specifically trained to perform translations from German (i.e., L2) to Italian (i.e., L1). Furthermore, in order to compensate for the small sample of subjects measured, I carefully selected the participants in such a manner as to estimate a putative influence of training experience and age on brain activity. Thereby, I measured two SIs with comparable age but differing in the number of training years (i.e., 33/31 years of age and 8/4 years of training, respectively), two subjects with similar age and years of training (i.e., 46/50 years of age and 22 years of training), as well as a subject situated in the middle of the sample distribution regarding age and training experience (i.e., 38 year of age and 14 years of training). Finally, it is important to mention that I consciously abstained from additionally measuring bilingual control subjects, since they are not able to perform simultaneous translations for a long time, leading to contamination effects that are driven by task difficulty, effort, and inability to perform the task.

Group analyses revealed that pars triangularis can be considered as a “hub” supporting backward language translation, whereas the left anterior insula seems to be more strongly involved in forward translation. Notably, single-subjects analyses revealed a somewhat different picture. In fact, even though the left pars triangularis was consistently activated in all five participants during backward translation, only three out of five participants showed increased activity in the insula (and only one out of them was characterized by a leftward asymmetry) during forward translation. These results lead to suggest that only pars triangularis can be considered as a “hub” region of the language-control network, whereas all other regions showing a remarkable inter-individual variability should more likely be considered as being “supportive” areas. In turn, I will discuss the functional role of these regions in more details by focusing on both commonalities as well as inter-individual variability.

### Functional commonalities in backward translation (L2 to L1): Broca's region

Results demonstrated consistent and robust brain activity within pars triangularis during backward translation compared to shadowing. Since this specific activation pattern emerged in each of the five subjects as well as from the group analysis, I propose that pars triangularis constitutes the “hub” region underling simultaneous language translation. Comparable results have previously been reported by Rinne and colleagues during both backward- and forward translation of complex sentences in SIs (Rinne et al., 2000). However, in this previous work the authors used PET instead of fMRI (suboptimal spatial and temporal resolution) and measured Finnish SIs specifically trained to translate in both language directions, namely from L2 to L1 as well as from L1 to L2. Most interestingly, a recent longitudinal fMRI study conducted with trainee SIs (Hervais-Adelman et al., 2015a) did not reveal increased brain responses in pars triangularis at T1 compared to T0 as a function of training. However, this is not really surprising, than the control group was composed of multilinguals, a specific sample of subjects that has previously been shown to activate the same brain region during both translation at the word- (Klein et al., 1995) and sentence (Hervais-Adelman et al., 2015b) level as well as in the context of language switching (Price et al., 1999; Quaresima et al., 2001; Garbin et al., 2011).

Continuous language switching over time constitutes a fundamental cognitive operation underlying working memory functions. Consequently, I propose that pars triangularis serves as a “hub” region supporting verbal working memory functions during simultaneous language translation. Certainly, the exact operationalization of the cognitive operations involved have to be elucidated in more details, than at the moment it still remains an open question whether this regions supports language switching mechanisms *per se*, domain-general working memory functions, or rather some other forms of cognitive control mechanisms, like attention or inhibition (or even a combination of these functions).

A further alternative explanation is that the consistent activity revealed within pars triangularis during backward translation reflects automaticity in language switching mechanisms. This point of view is strenghtened by the fact that the translation task I used in the present work was quite simple and easy to manage for professional SIs. Furthermore, similar brain responses could not be observed during forward translation, a task that has not been explicitely trained by the SIs measured and was therefore not automatized. Finally, based on previous studies showing a relationship between grays matter volume in the left pars triangularis and the cumulative number of training hours in SIs (Elmer et al., 2014a), data are interpreted as suggesting that automatic switching mechanisms are shaped by training.

### Functional commonalities in forward translation (L1 to L2): left anterior insula

Interestingly, even though group analyses clearly revealed that the anterior part of the left insula constitutes a common neural substrate of simultaneous language translation, this specific brain region was activated above threshold in three participants only. In addition, two out of these three subjects showed right-sided activity. The somewhat differential results between single-subject- and group analyses emphasize the importance of additionally examining functional distribution of brain activity within the single participants instead of only focusing on group statistics.

Previous work on simultaneous language interpretation has postulated that forward translation constitutes a more demanding task than backward translation (Klein et al., 1995; Rinne et al., 2000), from both cognitive- and articulatory perspectives. Therefore, I suggest that activity in the left anterior insula reflects articulatory- and cognitive operational demands while switching from L1 to L2. Based on the fact that none of the participants was a simultaneous bilingual, and on previous neuroimaging studies pointing to increased activity in brain regions supporting the motor act of speech in late- compared to early bilinguals (Frenck-Mestre et al., 2005), results are interpreted as reflecting stronger demands on articulatory- and sensory-to-motor coupling systems while translating sentences from the mother tongue into the weaker language. Certainly, future studies looking at the functional recruitment of the left anterior insula in overt speech production in subjects varying in the degree of speech competence are strictly required in order to confirm these findings.

Finally, it is important to mention that a previous meta-analysis focusing on the functional connectivity of the insula when participants were involved in active tasks (Cauda et al., 2012) revealed that the anterior part of this brain region is connected with frontal, cingulate, and parietal areas, and is mostly activated by cognition. Otherwise, the posterior portion of the insula was characterized by a more local connectivity pattern and connected to sensorimotor, temporal and posterior cingulate areas and rather related to interoception, perception, and emotion (for an extensive review consider Bamiou et al., 2003).

### Inter-individual variability in backward and forward translation

Single-subject analyses clearly demonstrated a huge range of intra-individual variability in the recruitment of “supportive” areas within the language-control network. This variability is interpreted as reflecting individual strategies during simultaneous translation that are possibly driven by several factors like experience, age of commencement, as well as predisposition. In turn, I will discuss the functional-anatomical architecture of these “supportive” areas by focusing on those brain regions that have not yet been discussed in the previous section, namely the basal ganglia, the middle-anterior cingulate cortex, and the inferior parietal lobe.

#### Caudate nuclei

Interestingly, there is evidence showing functional and structural differences in the caudate nuclei between bilinguals and monolinguals (van Heuven et al., 2008; Zou et al., 2012b; Abutalebi et al., 2013) as well as between professional or trainee SIs and multilingual control subjects (Elmer et al., 2011a, 2014a; Hervais-Adelman et al., 2015a). In addition, in a previous morphometric study of our group we provided evidence for a relationship between gray matter volume in the bilateral caudate nuclei and the cumulative number of practice hours, leading to suggest training-related adaptations. In the present work, I only observed sporadic evidence for a contribution of the caudate nuclei to simultaneous language translation. In fact, this was only the case for two subjects during backward translation (1 subject showed bilateral and another right-lateralized activity) and for one participant during forward translation (left lateralized). Consequently, the caudate nuclei cannot be considered as a “hub” region supporting language switching and translation mechanisms. Therefore, I propose that the caudate nuclei are rather subordinated to pars triangularis and recruited depending on individual-specific cognitive and motor demands.

#### Middle-anterior cingulate cortex

The present work did not reveal consistent activations in the middle-anterior cingulate cortex during backward- or forward or translation. In fact, during the more simple backward condition this region was only responsive in two subjects, whereas during forward translation a similar pattern emerged in three out of five subjects. Consequently, the middle-anterior cingulate cortex doesn't seem to constitute a “hub” area within the language-control network, but probably rather undertakes cognitive control mechanisms during high-load sentence processing. This line of argumentation is partially supported by a recent study of Hervais-Adelman and colleagues who revealed increased activity within the cingulate cortex in trainee SIs at T1 compared to T0 (Hervais-Adelman et al., 2015a). However, the cluster was not situated within the so-called “cognitive subdivision” of the cingulum (Bush et al., 2000) but rather just beyond it. By contrast, Rinne et al. (2000) used the same baseline condition as I used in the present study, namely shadowing, and did not revealed activity within this specific brain region at the group-level.

#### Inferior parietal lobe

In the present study, results only revealed sporadic activity within this specific brain region during both backward- (2 subjects bilaterally and one subject in the right hemisphere) and forward (one subject bilaterally and one in the right hemisphere) translation. Interestingly, none of the previous studies conducted with professional- (Rinne et al., 2000) or trainee SIs (Hervais-Adelman et al., 2015a) found distinctive activity within the inferior parietal lobe during simultaneous translation. In addition, in the context of single-words translation two previous studies reported even decreased activity (Price et al., 1999) or did not find significant activations in bilinguals (Klein et al., 1995).

## Limitations

In the present work, I performed statistical analyses on a small sample of professional SIs. Even though results consistently revealed left pars triangularis activation in all five participants, the sample measured here is obviously too small for drawing ultimate conclusions about the brain regions essentially involved in supporting cognitive control. Consequently, future studies using a similar experimental approach but with larger sample size are strictly required for drawing more robust conclusions about “hub” and “supportive” areas of the language control network during simultaneous translation. In addition, it is important to mention that all results have been reported with a *p*-value of 0.01 and were not corrected for multiple comparisons. Consequently, future studies using more powerful designs may be helpful in order to replicate the results reported in the present work. Finally, even though the investigation of SIs consistently translating in a solely language direction constitutes a fruitful approach for future research, this approach has the shortcoming of putative language-specific contaminations.

## Conclusions

In the present work, I tried to use an alternative approach and conjointly focused on group- and single-subjects analyses in order to disentangle “hub” and “supportive” areas of the language-control network during simultaneous language translation. Results indicated that only left pars triangularis was consistently activated across subjects. Consequently, results are interpreted as suggesting that only pars triangularis can be considered as a “hub” region, whereas all other areas of the control network are more likely susceptible to inter-individual variability and should therefore rather be considered as “supportive” regions. These results challenge previous models of language control mechanisms and may lay the base for a re-definition of the language-control network. The next important step will be to focus on functional connectivity between pars triangularis and the other areas of the control network in large samples of subjects in order to better comprehend the hierarchical dynamic interplay between “hub” and “supportive” areas.

## Author contributions

SE designed the study, performed the fMRI measurement, evaluated the data, and drafted the manuscript.

### Conflict of interest statement

The author declares that the research was conducted in the absence of any commercial or financial relationships that could be construed as a potential conflict of interest.
